# Unlocking the Potential of Immune Checkpoint Inhibitors in HR+/HER2− Breast Cancer: A Systematic Review

**DOI:** 10.3390/cancers17172940

**Published:** 2025-09-08

**Authors:** Giuseppe Di Grazia, Arianna Dri, Angela Grieco, Claudia Martinelli, Michela Palleschi, Federica Martorana, Giacomo Barchiesi, Grazia Arpino, Carmine De Angelis, Michelino De Laurentiis, Lucia Del Mastro, Fabio Puglisi, Paolo Vigneri, Mario Giuliano

**Affiliations:** 1 School of Specialization in Medical Oncology, Department of Human Pathology “G. Barresi”, Azienda Ospedaliera Universitaria Policlinico “G. Martino”, University of Messina, Via Consolare Valeria N. 1, Gazzi, 98125 Messina, Italy; 2 Department of Medicine (DMED), University of Udine, 33100 Udine, Italy; 3 Department of Medical Oncology, IRCCS Centro di Riferimento Oncologico (CRO) di Aviano, 33081 Aviano, Italy; 4 Department of Clinical Medicine and Surgery, University of Naples Federico II, 80131 Naples, Italy; 5 Medical Oncology, Breast & GYN Unit, IRCCS Istituto Romagnolo per lo Studio dei Tumori (IRST) “Dino Amadori”, 47014 Meldola, Italy; 6 Department of Clinical and Experimental Medicine, University of Catania, Torre Biologica “F. Latteri”-lato nord-p. 9 via Santa Sofia, 89, 95123 Catania, Italy; 7 Humanitas Istituto Clinico Catanese, University Oncology Department, Contrada Cubba Marletta, 11, Misterbianco, 95045 Catania, Italy; 8 UOC Oncologia A, Azienda Ospedaliero-Universitaria Policlinico Umberto I, 00161 Roma, Italy; 9 Scuola Superiore Meridionale, Via Mezzocannone, 4, 80134 Napoli, Italy; 10 Istituto Nazionale Tumori IRCCS Fondazione G Pascale, 80131 Naples, Italy; 11 IRCSS Ospedale Policlinico San Martino, UO Breast Unit, 16121 Genoa, Italy; 12 Department of Internal Medicine and Medical Specialties (DIM), Università di Genova, 16132 Genoa, Italy

**Keywords:** immune checkpoint inhibitors, HR+/HER2− breast cancer, clinical trials, biomarkers

## Abstract

**Background:** Hormone-receptor-positive (HR+)/HER2-negative (HER2−) breast cancer (BC) is characterized by low immunogenicity and an immunosuppressive microenvironment. These features likely contribute to the inconsistent clinical activity of immune checkpoint inhibitors (ICIs) in this BC subtype. We conducted a systematic review of clinical trials evaluating ICIs in HR+/HER2− BC patients, focusing on potential biomarkers of response and resistance to these drugs. **Methods:** We systematically searched in Medline via PubMed, EMBASE, and CENTRAL for phase II/III clinical trials published between 2013 and 2023, testing ICIs alone or in combination with other agents in HR+/HER2− BC patients at any stage. All the searches were performed up to 27 January 2024. Data on study characteristics, clinical outcomes, and biomarker profiles were extracted, and due to study heterogeneity, a narrative synthesis was performed, without risk-of-bias assessment or meta-analysis. **Results:** Twenty-five studies were included, with 3298 patients enrolled overall. Eighteen of these trials enrolled patients with advanced disease. All trials investigated ICI combination regimens, more frequently with chemotherapy, CDK4/6 inhibitors, or other immunotherapeutic agents. Most of the studies enrolling patients with advanced disease failed to show a significant clinical activity of ICIs. In the early setting, neoadjuvant chemo-immunotherapy with nivolumab or pembrolizumab increased the rate of complete responses compared to chemotherapy alone. Moreover, high programmed death-ligand 1 (PD-L1) expression, low ER (estrogen receptor), and high tumor-infiltrating lymphocyte (TIL) levels correlated with improved outcomes. Consistently, markers indicating enhanced immune activation, such as the MammaPrint High 2 (MP2) genomic signature, were associated with increased ICI sensitivity. **Discussion:** Despite the limited overall efficacy, ICIs may represent a viable therapeutic option for a selected subset of HR+/HER2− BC patients. However, this systematic review is limited by study heterogeneity and the inclusion of ongoing or immature trials, which prevents quantitative analysis and may affect future conclusions on ICIs in HR+/HER2− breast cancer. Finally, optimized combination strategies could enhance tumor immunogenicity, while predictive biomarkers such as PD-L1, TILs, or specific genomic signatures could identify responsive patients.

## Simple Summary

Immune checkpoint inhibitors (ICIs) have shown limited and inconsistent clinical activity in hormone-receptor-positive (HR+)/HER2-negative HER2− breast cancer, likely due to the low immunogenicity and immunosuppressive tumor microenvironment of these tumors. In advanced disease, clinical trials have reported modest efficacy of ICIs, with limited survival benefit. In the early setting, the addition of nivolumab or pembrolizumab to neoadjuvant chemotherapy has recently led to increased pathological complete response (pCR) rates, suggesting potential benefit in selected patients. Currently, several trials are evaluating novel combinations of ICIs with CDK4/6 inhibitors and other targeted therapies. While these approaches are promising, concerns remain regarding safety, tolerability, and real clinical efficacy. Moreover, the lack of predictive biomarkers limits patient selection and may contribute to inconsistent results across studies. Identifying reliable biomarkers of response is essential to optimize the use of immunotherapy in HR+/HER2− breast cancer and to tailor treatment strategies to those most likely to benefit.

## 1. Introduction

Breast cancer (BC) has been historically considered a scarcely immunogenic neoplasm. However, relevant heterogeneity exists in terms of immune responsiveness among BC molecular subtypes [1]. The type and extent of immune infiltrate vary greatly among BC subtypes and even within the same subtype [1,2]. Given these discrepancies, immunotherapy exhibits different degrees of efficacy according to BC biological features [3].

So far, triple-negative BC (TNBC) is the only subtype where immunotherapy has found clinical application. In several countries, the combination of chemotherapy with an immune checkpoint inhibitor (ICI) (atezolizumab or pembrolizumab) is the standard first-line treatment for patients with advanced TNBC whose cancer expresses programmed death-ligand 1 (PD-L1), based on the results of phase III randomized trials IMpassion130 and KEYNOTE-355 [4]. In patients with non-metastatic TNBC who are candidates for neoadjuvant therapy, pembrolizumab is routinely added to chemotherapy based on the results of the KEYNOTE-522 study [5].

Hormone-receptor-positive (HR+)/HER2-negative (HER2−) is the least immunogenic BC subtype. Initial attempts to use ICIs yielded disappointing results, probably due to the immunophenotypic profile of these tumors [1,6,7]. Indeed, HR+/HER2− BC usually harbors a relatively low tumor mutational burden (TMB), compared to TNBC. This determines a scarce neoantigen production and hinders the generation of an effective anti-tumor response [1]. A higher prevalence of immunosuppressive cells, such as regulatory T cells (Tregs) and myeloid-derived suppressor cells (MDSCs), coupled with low levels of tumor-infiltrating lymphocytes (TILs) has also been observed in HR+/HER2− BC [1].

However, combining immunotherapy with other agents capable of modulating the immune environment may overcome these barriers and enhance ICI efficacy [8]. Combinations with chemotherapy can induce immunogenic cell death and increase immune activation. Targeted therapies, such as cyclin-dependent kinase 4/6 inhibitors (CDK4/6is) and endocrine treatments, can modify the immune microenvironment by increasing programmed death-ligand 1 (PD-L1) expression and reducing immunosuppressive cell populations [8,9,10].

Altogether, these biological insights provide a strong rationale for exploring ICIs in HR+/HER2− BC despite its relatively “cold” immune phenotype. In particular, selected combinations with chemotherapy, endocrine therapy, or targeted agents may convert this subtype into a more immune-responsive disease. Systematic and timely evaluation of clinical evidence could help to define whether such strategies translate into meaningful clinical benefit.

We conducted a systematic review of the available evidence from phase II/III clinical trials testing ICIs in HR+/HER2− BC patients to recapitulate the latest advancements and highlight the challenges and the potential future developments of immunotherapy in this BC subtype.

## 2. Methods

This systematic review was realized according to the updated Preferred Reporting Items for Systematic Reviews and Meta-Analyses (PRISMA) guidelines [11]. The protocol was not prospectively registered in PROSPERO or any other systematic review registry.

We searched for records, published between 2013 and 2023, reporting the results of phase II and III clinical trials evaluating efficacy and safety of ICIs—alone or combined with other agents—in patients with HR+/HER2− BC at any stage. Phase I/II trials were included only if the results of the phase II part could be extrapolated.

To this purpose, the following terms and their synonyms and acronyms were variously combined in a search string: breast cancer, hormone receptor positive, immune checkpoint inhibitor, programmed death 1, programmed death ligand 1, cytotoxic T-lymphocyte antigen-4, nivolumab, pembrolizumab, atezolizumab, durvalumab, avelumab, cemiplimab, dostarlimab, spartalizumab, toripalimab, ipilimumab, tremelimumab, trial.

Full-length articles and published conference abstracts were considered. Reviews, editorials, commentaries, and case reports/series were excluded. Only publications written in English were eligible. Literature search for full-length articles was performed in Medline via PubMed, EMBASE, and CENTRAL. Abstracts were retrieved from the American Association of Cancer Research (AACR), American Society of Clinical Oncology (ASCO), European Society of Medical Oncology (ESMO), ESMO Breast Cancer, and San Antonio Breast Cancer Symposium (SABCS) congresses websites, and an individual search was carried out for each ICI. Additionally, a search for ongoing clinical trials was performed at Clinicaltrials.gov. All the searches were performed up to 27 January 2024.

After duplicate removals, two authors (GDG and AD) independently screened the titles and abstracts of each record. Controversies were resolved by a third author (FM). The full texts of selected records were then assessed for eligibility using the same procedure. Abstracts were separately screened by two authors (AG and CM), while another author (MP) assessed ongoing clinical trials’ eligibility.

Trials’ features and results, including efficacy and safety outcomes, were independently extracted in an electronic database by two authors (GDG and AD). For trials conducted in the metastatic setting, data on objective response rate (ORR) and survival were collected, while in the early setting, we focused on pCR and survival outcomes. Trials that did not stratify these endpoints by HR status were excluded, to ensure that only evidence specific to HR+/HER2− BC was included. Additionally, we explored data on potential biomarkers of interest reported in the included studies to identify patient subgroups more likely to benefit from ICI-based therapies.

Due to the vast heterogeneity of the included studies, a narrative synthesis of the available evidence was conducted to summarize and report the findings rather than a quantitative analysis (meta-analysis); moreover, we did not perform risk-of-bias assessment using standardized tools (e.g., Cochrane Risk of Bias or ROBINS-I), as their applicability across such a broad and heterogeneous set of studies would have been limited and potentially misleading.

## 3. Results

**Figure 1:**
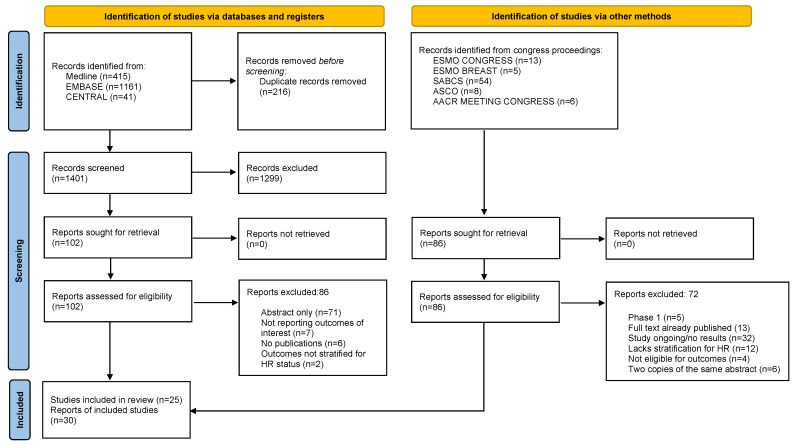
Flow diagram of included reports according to the Preferred Reporting Items for Systematic Reviews and Meta-Analyses (PRISMA). ESMO, European Society of Medical Oncology; SABCS, San Antonio Breast Cancer Symposium; ASCO, American Society of Clinical Oncology; AACR, American Association of Cancer Research; HR, hormone receptor.

A total of 1401 full-length articles and 85 abstracts were retrieved. A total of 1299 papers were excluded because they did not match the inclusion criteria. After screening and eligibility assessment, 30 reports (16 full-length articles and 14 abstracts) summarizing the results of 25 studies were included (Figure 1).

All but two trials were phase I/II or II, and most of them (n = 17, 68%) were non-randomized. Seventeen studies (68%) included only HR+/HER2− BC patients, whereas eight trials (32%) enrolled women with HER2− BC, including both HR+ and triple negative BC. Eighteen trials (72%) enrolled patients with advanced disease (Table 1), while the remaining seven (28%) were conducted in the neoadjuvant setting, with (n = 2) or without (n = 5) post-neoadjuvant extension of immunotherapy (Table 2).

The included studies enrolled a total of 3298 patients, 768 (23%) with advanced BC (ABC) and 2530 (77%) with early disease.

**Figure 2:**
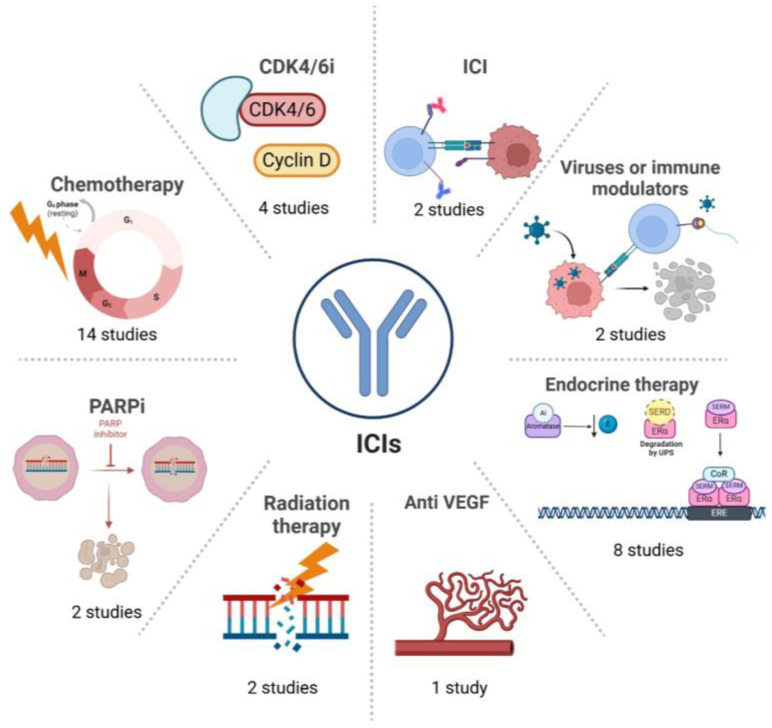
Combinations of immune checkpoint inhibitors tested in phase II/III trials enrolling HR+/HER2− BC patients. Some of the trials tested multiple combinations (e.g., ICI + CDK4/6is + endocrine therapy); therefore, the sum of combinations reported in this figure exceeds the number of included studies. ICI: immune checkpoint inhibitor, AI: aromatase inhibitor, E: estrogen, SERD: selective estrogen receptor degrader, ERα: estrogen receptor alpha, SERM: selective estrogen receptor modulator, CoR: co-receptor, VEGF: vascular endothelial growth factor, PARPi: poly-ADP ribose polymerase inhibitor, CDK4/6i: cyclin-dependent kinase 4/6 inhibitor.

All the studies evaluated the combination of a programmed death-1 (PD-1)/PD-L1 inhibitor with other agents, as detailed in Figure 2.

Pembrolizumab was the most investigated ICI (n = 11, 44%), followed by nivolumab (n = 7, 28%), durvalumab (n = 3, 12%), atezolizumab, and avelumab (n = 2, 8%, each). Two trials tested an ICI-ICI combination (i.e., nivolumab + ipilimumab and durvalumab + tremelimumab).

A biomarker analysis was reported in 15 studies (60%), including the evaluation of PD-L1 expression, tumor-infiltrating lymphocytes (TILs), TMB, gene expression signatures, or other markers. However, none of the included trials selected patients according to PD-L1 status or other specific immune-related biomarkers.

### 3.1. ICIs and Chemotherapy

Fourteen trials tested ICIs in combination with chemotherapy, nine in the advanced setting and five in patients with non-metastatic BC.

In three studies, chemotherapy and ICIs were combined with other treatments, such as adjuvant endocrine therapy (two studies), a poly-ADP ribose polymerase (PARP) inhibitor (one study), another ICI (one study), an anti-vascular endothelial growth factor (VEGF) (one study), and radiotherapy (RT) (one study). Taxanes were the most frequently used chemotherapeutic agents, either alone or in combination with other treatments (nine studies), followed by anthracyclines (six studies), eribulin (three studies), carboplatin (one study), and capecitabine (one study).

Mono-chemotherapy with a taxane in combination with an ICI or with an ICI and bevacizumab showed signals of efficacy in three trials in the advanced setting, while in the neoadjuvant setting, the combination of anthracyclines and taxanes with immunotherapy significantly improved the rate of pathological complete response (pCR) [20,26,27,31,35,36]. The TATEN trial was a single-arm study of pembrolizumab and paclitaxel in CDK4/6i-pretreated/chemotherapy-naïve HR+/HER2− ABC patients with a non-luminal disease according to PAM50 profiling. Results of an interim analysis from 15 patients showed an ORR of 53.3% [95% confidence interval (CI), 26.6–78.7], a CBR of 86.6% (95% CI, 59.5–98.34), and a median PFS of 7.5 months (95% CI, 5.6–10.2). Of note, the rate of G3 adverse events (AEs) was 53.5% [27]. In the phase II NEWBEAT trial, the triple association of nivolumab, paclitaxel, and bevacizumab was tested as first-line therapy in HER2− ABC patients. Among HR+/HER2− patients (n = 39), objective responses were observed in 74% of cases, with a median PFS of 16.1 months. Neither PD-L1 status nor VEGF expression correlated with outcomes. G3 and G4 AEs occurred in 53% and 5% of patients, respectively [20]. Another single-arm study (NCT02752685) tested pembrolizumab and nab-paclitaxel in HR+/HER2− ABC patients who received up to two previous chemotherapy lines in the advanced setting. Five out of twenty patients (25%) achieved a partial response (PR), with a median DOR of 3.9 months (95% CI, 2.07–not reached), while seven (35%) patients had disease stabilization. Median PFS (mPFS) was 5.6 months (95% CI, 2.07–8.18), and median OS (mOS) was 15.7 months (95% CI, 3.88–27.70). G3 AEs occurred in 70% of patients [26].

Anthracyclines and cyclophosphamide combined with an ICI showed modest efficacy both in early and advanced settings. In a trial enrolling patients with HR+ ABC, treated with at least one previous line of chemotherapy, the addition of nivolumab and ipilimumab to pegylated liposomal doxorubicin (PLD) and cyclophosphamide did not show any improvement in PFS, ORR, and CBR. A numerical PFS advantage emerged in patients with a Treg gene signature, while outcomes did not vary according to PD-L1 status and TMB [37]. However, the rate of AEs was significantly higher in the combination arm (63% vs. 39%). In the phase Ib/II single-arm B-IMMUNE study, durvalumab was added to neoadjuvant dose-dense epirubicin + cyclophosphamide (EC) after 12 infusions of weekly paclitaxel. Eight (33%) of the twenty-five HR+/HER2− patients achieved a pCR. Grade 3 AEs occurred in 14.6% of patients, whereas immune-related AEs were reported in four (16.0%) patients [32].

Eribulin combined with an ICI (pembrolizumab or nivolumab) showed conflicting evidence of efficacy in three trials in advanced BC (aBC). Pembrolizumab and eribulin were combined in a single-arm trial (KELLY), with an ORR of 40.9% and a clinical benefit rate (CBR) of 56.8% (95% CI, 41.0–71.7) in the overall population. The benefit was maintained regardless of PD-L1 status. Moreover, grade 3 (G3) and grade 4 (G4) adverse event (AE) rates were 56.8% and 25.9%, respectively [22]. By contrast, the addition of pembrolizumab to eribulin did not provide any significant ORR and progression-free survival (PFS) advantage compared to eribulin alone in a randomized phase II study including 88 pretreated HR+/HER2− aBC patients (NCT03051659). Even in this case, PD-L1 status, TILs, and tumor mutational burden (TMB) did not show a significant association with outcomes. Furthermore, toxicity in the eribulin arm was similar to the control one, with G3 AEs reported in 68% and 61% of patients, respectively. [7]. A third trial (KCSG BR18-16) evaluated nivolumab and eribulin in pretreated HER2− aBC Asian patients. In the HR+ cohort, median PFS was 5.6 months (95% CI, 5.3–7.4), and the 6-month PFS rate was 47.2% (95% CI, 34.4–64.8). Confirmed ORR was 35.6% and median duration of response (DOR) was 6.9 months (95% CI: 5.6–18.3). No association of PD-L1 status with ORR emerged. G3 AEs were reported in 65.6% of patients [17].

In the PEMBRACA trial, the combination of pembrolizumab and carboplatin did not show any sign of activity in a selected population with a known germline BRCA1/2 pathogenic variant. No new safety signals were reported in this trial, with a G3 AE rate of 22.3% [25].

Similarly, no benefit was reported when pembrolizumab was added to capecitabine in 14 endocrine-refractory HR+/HER2− ABC patients pretreated with one to two treatment lines. The safety profile was consistent with previous studies [21].

The promising activity of taxanes and anthracyclines with ICIs translated into a greater improvement of their efficacy when combining these chemotherapeutic agents in standard early BC (EBC) in several trials. One arm of the phase II I-SPY2 platform trial compared pembrolizumab plus neoadjuvant chemotherapy (NACT) to NACT alone in stage II/III HER2− BC patients (HR+ n = 40). Pathological complete responses were more than double in patients receiving pembrolizumab (30% with pembrolizumab + NACT versus 13% with NACT alone). Moreover, patients who did not achieve a pCR had lower residual cancer burden (RCB) scores when treated with pembrolizumab + NACT as compared to NACT alone. However, no significant difference in event-free survival (EFS) was found between treatment arms [31].

Given these encouraging results, two large multicenter, randomized, placebo-controlled trials investigated the addition of a PD-1 inhibitor to standard NACT and its extension in the post-neoadjuvant phase. In the KEYNOTE-756 trial, 1278 patients with cT1c-2 cN1-2 or cT3-4 cN0-2, grade 3 ER+/HER2− BC were enrolled. They were randomized 1:1 to standard-of-care NACT (weekly paclitaxel for 12 cycles followed by anthracyclines plus cyclophosphamide for 4 cycles) plus pembrolizumab or placebo, followed by up to 9 additional cycles of pembrolizumab/placebo plus endocrine therapy after surgery. Primary endpoints were pCR and EFS. In the intention-to-treat (ITT) population, the addition of pembrolizumab to NACT showed a statistically significant improvement in pCR rate [24.3% (95% CI, 21.0–27.8) in experimental arm vs. 15.6% (95% CI, 12.8–18.6) in control arm]. This difference was maintained across all the pre-specified subgroups. Of note, ER-low patients experienced a 25.6% increase in pCR rates from the addition of pembrolizumab to NACT, compared to 8% in patients with ER 10–100%. Grade 3 treatment-related AEs were more frequent in the experimental arm (52.5% vs. 46.4%), but no new safety concerns were reported [36].

The second phase III trial (Checkmate-7FL) randomized 510 early-stage (stage II-III, G3 with ER ≥ 1% or grade 2 with ER 1–10%) ER+/HER2− BC patients to standard NACT plus nivolumab or placebo. Also, in this case, nivolumab/placebo administration was continued after surgery for up to seven cycles, along with adjuvant endocrine therapy. Following a protocol amendment, the primary endpoint focused solely on pCR, whereas EFS was included as an exploratory endpoint. The study was positive as pCR rate was significantly higher in the experimental arm (24.5% vs. 13.8%; OR 2.05, 95% CI, 1.29–3.27; *p* = 0.0021). Residual cancer burden (RCB) 0-I rates were also higher in the nivolumab arm (30.7%, 95% CI, 25.2–36.8 vs. 21.3%, 95% CI 16.5–26.9). The pCR improvement was more evident among patients with stage III (ORR 30.7% vs. 8.1%) and PD-L1+ disease (ORR 44.3% vs. 20.2%). Moreover, pCR rate increased in poorly differentiated ER-low (ER-1–10%) and in ER ≤ 50% patients compared to ER > 50%, with a delta of 27%, 29.3%, and 7.4%, respectively, with the control arm. Similarly, a higher increase in pCR rate was found in G3 tumors with PR < 10% expression. G3/4 AE incidence was similar (35% vs. 32%) across the study arms, with a higher prevalence of immune-related AEs in the nivolumab group [35].

Regarding the safety, distinct patterns of AEs emerged for different combinations.

As expected, hematologic toxicities were the most common with ICI and chemotherapy, regardless of the specific regimen. Neutropenia, anemia, and thrombocytopenia were frequently observed, often requiring dose reduction or interruption.

Non-hematologic toxicity and irAEs were generally of low grade, with endocrinopathies, especially thyroid dysfunction, representing the most frequent toxicity. Grade ≥ 3 irAEs were rare, and treatment discontinuations or dose modifications were predominantly due to chemotherapy-related toxicities. Overall, the combination of ICI and chemotherapy demonstrated a manageable safety profile when patients were closely monitored and adverse events were addressed promptly.

### 3.2. ICIs and Endocrine Therapy with or Without CDK4/6 Inhibitors

The potential synergy between PD-1/PD-L1 inhibitors and endocrine therapy, with or without CDK4/6i, was explored in several studies. Pembrolizumab was tested with exemestane and gonadotropin-releasing hormone agonist (GnRHa) in a cohort of 14 chemotherapy-naïve premenopausal Asian patients with HR+/HER2− ABC. The objective response rate was 35.7%, while the PFS rate at 8 months was 64.3%. TILs, TMB, and PD-L1 expression were not associated with outcomes, although the level of dendritic and natural killer (NK) cell infiltration in pretreatment specimens correlated with the response, and immune cell populations increased in post-treatment tissue samples [28].

Three additional trials tested an ICI with a CDK4/6i. The first was a phase I/II single-arm non-randomized trial enrolling 16 patients treated with pembrolizumab, palbociclib, and letrozole in a first-line setting. Response rate was 56%, median PFS 25.2 months (95% CI, 5.3–not reached), and median OS 36.9 months (95% CI, 36.9–not reached). No difference in outcome emerged according to PD-L1 status, TILs, and genomic and transcriptomic analysis. G3 and G4 AE rates were 87% and 40%, respectively [24]. The PACE trial, which enrolled HR+/HER2− ABC patients progressing upon treatment with a CDK4/6i and an aromatase inhibitor, included a treatment arm with the combination of avelumab, palbociclib, and fulvestrant. Median PFS was numerically longer with the triplet (8.1 months, HR = 0.75; 90% CI, 0.50–1.12; *p* = 0.23) as compared to fulvestrant monotherapy (4.8 months) and fulvestrant plus palbociclib (4.6 months). The addition of the PD-L1 inhibitor also numerically improved ORR, which was 13.0% (90% CI 5.4–20.5%) with avelumab plus palbociclib and fulvestrant, 9.0% (90% CI 4.5–13.5%) with palbociclib and fulvestrant, and 7.3% (90% CI 1.5–13.0%) with fulvestrant alone [13]. Lastly, the association of nivolumab, abemaciclib, and endocrine therapy (letrozole or fulvestrant) was evaluated in the NEWFLAME trial. This study was prematurely discontinued due to an unacceptable toxicity rate, as 16/22 enrolled patients (73%) experienced grade ≥ 3 AEs, leading to permanent treatment discontinuation in 10/22 (45%) patients [19].

Immunotherapy plus endocrine therapy, with or without a CDK4/6i, was also evaluated in the pre-operative setting. In the phase II GIADA trial, nivolumab was combined with triptorelin and exemestane after three cycles of anthracycline-based NACT in premenopausal HR+/HER2− EBC patients. pCR was achieved in 7 (16.3%, 95% CI 7.4–34.9) of the 43 women enrolled. The most frequent AEs of any grade were increased liver enzymes and arthralgia. The most frequently observed immune-related AEs were G1-2 endocrinopathies, involving thyroid and, less frequently, adrenal or hypophysis dysfunctions [33]. Higher pCR rates were observed in patients with basal BC subtype at PAM50 analysis (50%), as compared to luminal A (9.1%) and luminal B (8.3%) subtypes. Moreover, TIL levels were higher in patients with pCR (15%) compared to those without pCR (2%). Lastly, gene signatures associated with inflammation were overexpressed in the case of pCR.

The Checkmate 7A8 phase Ib/II study evaluated nivolumab plus palbociclib and anastrozole. Twenty-one patients with HR+/HER2− BC and primary tumor ≥ 2 cm were enrolled. Nine received palbociclib at the dose of 125 mg, and twelve at the dose of 100 mg. Despite the promising activity (ORR 66.7% and 75% in the 125 mg and 100 mg groups, respectively), this regimen determined a considerable rate of G3/4 hepatotoxicity (44.4% and 25% in the 125 mg and 100 mg groups, respectively), leading to treatment discontinuation in nine patients and to premature trial termination [34].

Overall, combinations of ICIs with endocrine therapy with or without CDK4/6i were associated with serious toxicity. While hematologic AEs (neutropenia, anemia, thrombocytopenia) were frequent but manageable, irAEs—hepatotoxicity, pneumonitis, and endocrinopathies—were of higher grade, often leading to treatment discontinuation or interruption [34]. Overall, endocrinopathies (hypothyroidism and adrenal insufficiency) represented the most common irAEs. Close monitoring of liver function tests, pulmonary status, and endocrine parameters was essential, and prompt initiation of corticosteroids or other immunosuppressive therapies facilitated recovery in most cases; however, some treatment-related deaths occurred, leading to trial premature termination [19,34].

### 3.3. Other Combinations

Several alternative therapeutic regimens have been investigated in HR+/HER2− ABC patients; however, none of these combinations provided substantial clinical benefit in an unselected population.

In one arm of the I-SPY2 trial, the triple combination of an ICI (durvalumab), a PARPi (olaparib), and NACT was evaluated. In further detail, durvalumab with olaparib was associated with weekly paclitaxel and then followed by four cycles of anthracyclines plus cyclophosphamide, compared to standard NACT, in 52 HR+/HER2− EBC patients. The pCR rate was 28% in the combination arm versus 14% in the standard arm. Moreover, patients treated with durvalumab and olaparib had lower RCB scores. G3 AEs occurred in 56% of patients receiving the combination versus 34% in those receiving standard chemotherapy; immune-related AEs occurred in 27.4% and 2% of patients, respectively. In this study, high ESR1 and progesterone receptor (PR) expression levels, along with a mast-cell gene signature, were associated with lack of pCR [30]. Finally, another trial evaluated PARPi, in particular talazoparib, in combination with avelumab, also showing initial signals of activity for the combination in the advanced disease [14].

The single-arm SOLTI-PROMETEO trial evaluated atezolizumab in combination with the oncolytic virus talimogene laherparepvec (T-VEC) in patients with residual disease after standard NACT, assessed by magnetic resonance imaging (MRI) and core biopsy. Among the 20 enrolled patients, seven (30%) achieved a pCR, but surprisingly, none of them had a radiological response (PD n = 2, SD n = 5). Grade 3 neutropenia occurred in one patient. Biomarker analyses on tumor samples showed an increase in TILs and immune PD-L1+ cells, as well as an increased expression of immune genes and after one dose of T-VEC, after one dose of the combination, and at surgery. Gene expression changes at surgery, compared to baseline, occurred in 73.1% of patients, with a predominant shift from luminal A/B to normal-like subtype [29].

Another innovative approach was evaluated in the MORPHEUS HR+ trial, where the combination of atezolizumab and entinostat (a selective inhibitor of histone deacetylase) did not show any benefit compared to fulvestrant, despite increasing the incidence of AEs [12].

Lastly, two small phase II trials tested the concomitant use of an ICI (nivolumab or pembrolizumab) with RT applied to metastatic sites in HR+/HER2− ABC patients. Despite this approach being safe, it did not show significant signs of activity [18,23].

Combinations of ICIs and other targeted agents, such as PARPi or experimental therapies, increased the incidence of AEs [29,30]. Conversely, the association of radiotherapy was generally safe [18,23]. Hematologic toxicities were frequent, while non-hematologic ones varied depending on the specific agent. Hepatotoxicity, skin rash, pneumonitis, and endocrinopathies were the most common irAEs, with some patients experiencing grade ≥ 3 events, often leading to treatment discontinuation. Management of these events often required treatment interruption and immunosuppressive therapy.

### 3.4. Ongoing Clinical Trials

A total of 17 ongoing trials were identified. Of these, 6 studies were conducted in the advanced and 11 in the early disease stage. Fourteen studies were phase II, and three were phase III. Sixteen studies explored a combination regimen incorporating an ICI, while one trial evaluated a monotherapy of a new IL-15 antibody fusion protein targeting CTLA-4. The most common tested combinations included chemotherapy (six studies), antibody–drug conjugates (ADCs) (three studies), PARPi (three studies), radiation therapy (three studies), another ICI (two studies), CDK4/6i (two studies), and endocrine therapy (two studies). The main features of the ongoing clinical trials are reported in Table 3.

### 3.5. Biomarkers of Response to ICIs in HR+/HER2− BC

**Figure 3:**
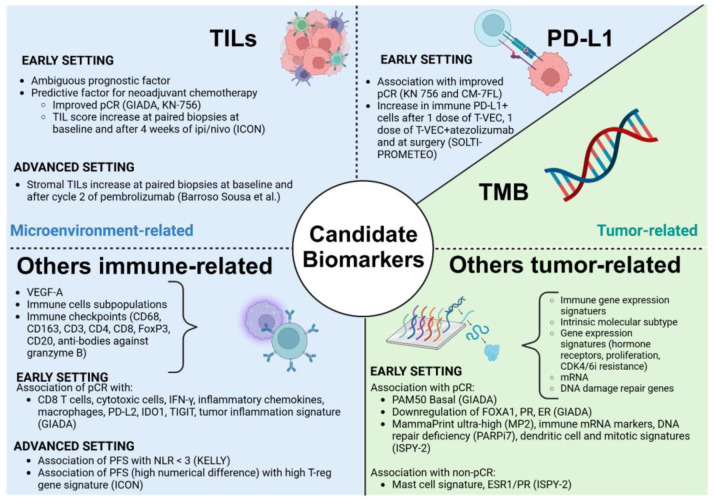
Candidate biomarkers of response to ICIs evaluated in the included studies. TILs: tumor-infiltrating lymphocytes, pCR: pathologic complete response, PD-L1: programmed death-ligand 1, T-VEC: talimogene laherparepvec, TMB: tumor mutational burden, CDK4/6i: cyclin-dependent kinase 4/6 inhibitor, NLR: neutrophil-to-lymphocyte ratio, PR: progesterone receptor, ER: estrogen receptor, PARPi: poly-ADP ribose polymerase inhibitor, ESR1: estrogen receptor 1, VEGF-A: vascular endothelial growth factor.

The identification of biomarkers capable of predicting response to ICIs in BC remains a key issue. Figure 3 illustrates biomarkers explored in the included trials, dividing them into tumor-related and microenvironment-related.

Programmed death-ligand 1 expression was associated with improved pCR in KN-756 and CM-7FL [35,36]. Moreover, in the SOLTI-PROMETEO trial, an increase in PD-L1+ cells was reported after one dose of T-VEC, after one dose of T-VEC + atezolizumab, and at surgery [29].

High levels of TILs represented a predictive factor of response to NACT in the GIADA and KN-756 trials, correlating with a higher probability of pCR [33,35]. Moreover, in the ICON trial, TIL score increased at paired biopsies from baseline and after 4 weeks of treatment with ipilimumab and nivolumab, in patients with target lesion reduction [16]. In the advanced setting, Barroso Sousa et al. showed an increase in stromal TILs at paired biopsies from baseline and after cycle 2 of pembrolizumab and palliative RT [23].

Despite being considered a promising biomarker in other tumors, no correlation between TMB and response to ICIs was reported in the studies included in our review.

Other microenvironment-related biomarkers were explored in the included trials. In the early setting, immune cell subpopulations (CD8 T cells, cytotoxic cells, macrophages), soluble mediators (IFN-γ, inflammatory chemokines), other immune checkpoint expression levels (PD-L2, IDO1, TIGIT), and tumor inflammation signature correlated with pCR in the GIADA trial [33]. On the other hand, in the advanced setting, neutrophil-to-lymphocyte ratio (NLR) < 3 was associated with higher mPFS in the KELLY trial [22]. Lastly, a high Treg gene signature correlated with a higher numerical difference in PFS in the ICON trial [16].

Regarding other tumor-related biomarkers, PAM50 basal BC and downregulation of FOXA1, PR, and ER genes were associated with pCR in the GIADA trial [31]. Moreover, Mammaprint ultra-high (MP2) BC, showing exceptionally high pCR rates in response to neoadjuvant durvalumab plus olaparib in the I-SPY2 trial, probably represents the most significant biomarker of response [28]. These results led to a currently ongoing phase III trial evaluating neoadjuvant ICIs plus chemotherapy, specifically in this subgroup of patients (NCT06058377). Furthermore, in the I-SPY2 trial, other signatures correlated with pCR, such as immune mRNA markers, DNA repair deficiency (PARPi), and dendritic cell and mitotic signatures [28]. Conversely, several biomarkers, such as mast-cell signature and ESR1/PR, showed an inverse association with pCR [30].

No significant correlation was found between a biomarker and the response to a specific type of treatment in the advanced setting, while in the early setting, PD-L1 expression, TILs, and MP2 signature represent the most promising predictive factors of response to neoadjuvant immunotherapy.

## 4. Discussion

Immunotherapy represents a field of intense clinical investigation in all tumor types, including BC. While ICIs, in combination with chemotherapy, are already standard of care in TNBC, incorporating these agents in the treatment of HR+/HER2− BC seems far more challenging [38]. To recapitulate the available evidence in this field, we reviewed the recently published phase II/III trials testing ICIs in HR+/HER2− BC, both in the advanced and early disease stages. To the best of our knowledge, this is the first systematic review focusing on this topic.

All the 25 selected trials tested an ICI combined with at least one other agent. The choice to test only combination regimens likely derives from the scarce immunological responsiveness of HR+/HER2− BC, which represents the immune “coldest” BC subtype [1]. Several preclinical and translational studies suggest that adding to ICIs a second agent, such as chemotherapy, CDK4/6i, or PARPi, may elicit an anti-tumor immune response, ultimately resulting in a synergistic effect [10,39,40].

Among chemotherapeutic drugs, taxanes provided the best performance when combined with ICIs in patients with HR+/HER2− BC, especially in the advanced setting. The reason for this difference in efficacy is still to be investigated; however, preclinical evidence showed an immunogenic effect of a low dose of paclitaxel in animal models of BC [41]. Evidence about the immunomodulatory effect of anthracyclines also exists. These compounds seem to stimulate the expression of the pattern recognition receptors (PRRs), toll-like receptor-3 (TLR3), and the secretion of type I interferons and chemokine CXCL10 [41]. In the TONIC trial, priming with doxorubicin in TNBC induced a more favorable tumor microenvironment and was correlated with a higher probability of response to nivolumab [42]. In the GIADA trial, changes in immune cell populations were observed after neoadjuvant treatment with anthracycline [33]. However, the combination of PLD, cyclophosphamide, and nivolumab did not show efficacy in HR+/HER2− ABC patients [16]. In the early setting, the large phase III trials KEYNOTE-756 and CheckMate-7FL demonstrated significant improvements in pCR rates with the addition of pembrolizumab or nivolumab to NACT, particularly in ER-low subgroups [35,36]. Whether these short-term results will translate into EFS or overall survival (OS) benefit is unknown, also considering the weaker prognostic role of pCR in HR+/HER2− compared to the other breast cancer subtypes [43]. Moreover, the benefit of neoadjuvant ICIs, as reported in both studies, came at the cost of increased toxicity and irAEs, underscoring the need to balance efficacy with long-term safety and feasibility in this patient population.

Despite encouraging preclinical data suggesting that CDK4/6i may improve tumor immunogenicity via multiple mechanisms, including the promotion of antigen presentation and T-cell activation, the suppression of Treg proliferation, and an increase in PD-L1 expression, clinical combinations with ICIs have not yielded meaningful results so far [39]. Possible explanations may include pharmacokinetic and pharmacodynamic interactions, with CDK4/6 inhibition potentially dampening T-cell proliferation, as well as suboptimal sequencing of therapy since concurrent administration may enhance toxicity without maximizing synergy. In fact, toxicity represents a major hurdle to the implementation of these regimens in the clinical practice, given the considerably high incidence of adverse events, including immune-related hepatotoxicity and interstitial lung disease (ILD)/pneumonitis [21,44]. Therefore, a potential future strategy could involve defining the optimal sequence of CDK4/6i and immunotherapy, leveraging their “immuno-priming” effect and improving safety at the same time.

Increasing evidence is available about the synergism between PARPi and immunotherapy, especially in carriers of germline BRCA1/2 pathogenic variants [40]. The rationale of this combination comes from the hypothesis that PARP inhibition in BRCA1/2 mutated cancer cells may elicit genomic instability, generating DNA fragments able to activate the intracellular stimulator of interferon genes pathway [40]. Hence, genomic instability might generate larger amounts of immunogenic neoantigens. Preclinical evidence showed that PARP inhibition can upregulate PD-L1 expression in breast cancer cell lines and animal models [40]. In this scenario, three clinical trials investigated the combination of a PARPi and an ICI in several tumors, including BC, such as MEDIOLA, TOPACIO, and DUO-O trials, showing initial signals of activity [45,46,47]. While this approach is in the advanced phase of development in ovarian cancer, results in HR+/HER2− BC are still immature and deserve further investigation, as shown by the results of the JAVELIN PARP Medley and I-SPY2 trials in the advanced and early settings, respectively [14,30].

Regarding the safety of the presented approaches, there is a critical need for proactive monitoring and management of toxicities. Baseline assessment of liver function and thyroid and adrenal axes and regular hematologic monitoring are recommended to detect early signs of irAEs or myelosuppression. Prompt intervention with corticosteroids or immunosuppressive agents is essential to manage grade ≥ 3 immune-mediated toxicities, particularly hepatotoxicity, pneumonitis, and severe endocrinopathies. Dose interruptions, reductions, or discontinuation should be considered according to the severity of the event, with multidisciplinary input from oncology, endocrinology, and hepatology specialists. Patient education regarding symptom recognition is crucial for timely reporting and management. These strategies are particularly relevant for multi-agent regimens, where overlapping toxicities may limit treatment tolerability and overall feasibility, highlighting the balance between maximizing efficacy and minimizing harm. However, as mentioned above, all these strategies might still not be sufficient to avoid potentially fatal toxicity for some treatment combinations (e.g., CDK4/6i) [44].

Among the strategies under investigation, the combination of ICIs and ADCs was one of the most promising [48]. Beyond their direct cytotoxicity, ADCs can enhance tumor immunogenicity by inducing immunogenic cell death, releasing tumor antigens and neoantigens, modulating the tumor microenvironment (e.g., reducing Tregs, upregulating PD-L1, activating cGAS–STING), and ultimately promoting CD8^+^ T-cell recruitment and activation [49,50,51]. This creates a more favorable immune context that may synergize with ICIs. Clinically, definitive results are still awaited: the phase II SACI-IO HR+ trial (sacituzumab govitecan ± pembrolizumab) was negative, while ongoing studies such as DESTINY-Breast08 (T-DXd + durvalumab) and TROPION-Breast04 (Dato-DXd + durvalumab) are expected to clarify the role of ADC–ICI combinations, which may vary depending on payload and linker design [52,53,54]. Beyond ADCs, several other approaches are being explored, including bispecific antibodies, adoptive cell therapies (CAR-T, TCRs, TILs), tumor vaccines, and novel checkpoint modulators. For instance, the LAG-3 agonist eftilagimod alpha showed modest benefit in the AIPAC trial, with some survival advantage in younger patients [55]. Bispecific antibodies, such as zanidatamab, have shown early efficacy in combination regimens, while therapeutic vaccines and adoptive T-cell therapies are under active investigation but remain experimental in HR+/HER2− BC [56].

Nonetheless, a major challenge in the field of immuno-oncology is the lack of reliable biomarkers for identifying the subset of patients who may truly benefit from ICIs, alone or in combination with other agents [9]. Many of the trials included in our review incorporated correlative analyses, yet consistent predictive markers remain elusive. Among the most studied biomarkers, PD-L1, TILs, and TMB are potentially implementable in clinical practice [57,58], though their predictive role has been inconsistent across settings. In advanced disease, PD-L1 expression showed no correlation with ICI efficacy (e.g., NEWBEAT, KELLY), whereas in early-stage trials (KEYNOTE-756, CheckMate-7FL), PD-L1 positivity was associated with higher pCR rates [59,60]. Importantly, the benefit of pembrolizumab and nivolumab was maintained across all subgroups but appeared larger in patients with ER-low tumors (1–10% ER expression). However, the definition of “ER-low” remains controversial, with varying cut-offs and assays across studies, highlighting the urgent need for standardized methodologies [61]. Biological evidence suggests that ER-low tumors may be more sensitive to immunotherapy since they share features with TNBC, including higher TIL levels, enrichment in basal-like subtypes, and immune-related gene expression [62].

Baseline TILs correlated with NACT response across BC subtypes, and they consistently showed predictive value in GIADA, KEYNOTE-756, and CheckMate-7FL [33,59,60]. However, this correlation was not observed in the advanced setting [7,13,17,21]. Unlike in TNBC or HER2-enriched BC, where high TILs predict improved survival, their role in luminal-like tumors remains equivocal, with some studies reporting an inverse association with OS [63,64]. Recent efforts have proposed potential cut-offs for PD-L1 positivity and TIL levels to assess their prognostic and predictive significance specifically in TN and HER2+ BC (e.g., ≥10% CPS for PD-L1, stromal TILs ≥ 10% or 20%) [65,66,67]. However, since no thresholds have been currently defined for HR+/HER2− BC, these biomarkers require prospective validation before clinical implementation.

Similarly, although TMB is an agnostic biomarker of response to ICIs [58], no predictive correlation was found in HR+/HER2− metastatic BC, suggesting that TMB alone may not be sufficient to guide patient selection in this subtype. Conversely, the MP2 genomic signature has emerged as a promising predictor of ICI sensitivity, with striking results in the neoadjuvant setting compared to Mammaprint high-1 (MP1) tumors [30]. Evidence from the I-SPY2 trial suggests potential threshold scores for identifying MP2 tumors, but ongoing trials (e.g., NCT06058377) are needed to confirm clinical applicability and reproducibility.

Dynamic biomarkers, such as early changes in circulating tumor DNA, immune cell subsets, or transcriptomic signatures, could refine patient selection beyond static baseline features. Nonetheless, costs and technical challenges limit their near-term applicability. Ultimately, identifying the responsive subset (e.g., ER-low, MP2 signature, inflamed phenotype) is paramount to balance the modest efficacy observed in advanced disease with the meaningful, but toxicity-burdened, benefits seen in the early setting. A careful risk–benefit assessment is therefore essential when considering ICI integration into HR+/HER2− BC management.

Lastly, recent evidence is emerging on the role of PD-1 gene polymorphisms and their potential impact on prognosis and response to ICIs in several malignancies, such as lung cancer and melanoma, adding another layer of complexity to the understanding of immunotherapy efficacy [68,69]. Metagenomic studies have shown that specific PD-1 variants may modulate immune checkpoint activity and influence treatment outcomes, suggesting a possible avenue for biomarker development. However, no studies to date have evaluated PD-1 polymorphisms in HR+/HER2− breast cancer, underscoring the need for further investigation before these findings are translated into clinical practice.

This systematic review has some limitations. The heterogeneity of the included studies did not allow performing a quantitative analysis. Additionally, some of the included studies are still ongoing and/or have immature results. Hence, while it is possible to imagine the comprehensive framework for the use of ICIs in HR+/HER2− BC, further development may alter this scenario in the near future.

## 5. Conclusions

According to the available evidence, ICIs may have a role in the treatment of selected HR+/HER2− BC patients, but several challenges still need to be addressed. In the metastatic setting, none of the attempts to incorporate ICIs into current standard therapies provided potentially practice-changing results. However, newer combinations, such as those with CDK4/6i or ADCs, may modify this scenario. In this context, proper management of toxicities will be paramount for the development of combination regimens.

In the early setting, the association of a PD-1 inhibitor with neoadjuvant chemotherapy is a promising strategy, especially in a selected high-risk population. In the future, it would be crucial to understand which patients would benefit the most from this approach and how to integrate ICIs with adjuvant CDK4/6i.

In this context, the identification of new predictive biomarkers will be essential for guiding the design of improved clinical trials and pursuing personalized immunotherapies for HR+/HER2− BC patients.

## Figures and Tables

**Table 1:** Phase II and III clinical trials evaluating ICIs in HR+/HER2− advanced breast cancer patients.

Study ID/Name	ICIs	Phase	Primary Endpoint	Study Design	Interventions (No. of pts)	Efficacy Outcomes	TRAEs G ≥ 3 (%)	Biomarkers
NCT03280563/ MORPHEUS-HR+ Breast Cancer [12] *Sonnenblick A et al.*	Atezolizumab	Ib/II	ORR	Open-label, randomized (1:1)	Atezolizumab + entinostat (n = 15) vs. fulvestrant (n = 14)	ORR 6.7% (95% CI 0.17–31.95%) vs. 0% (95% CI 0–23.16) mPFS 1.8 m (95% CI 1.5–3.6%) vs. 1.8 m (95% CI 1.5–2.7%)	40% vs. 21.4%	PD-L1
*NCT03147287/* *PACE* [13] *Mayer EL et al.*	Avelumab	II	PFS	Open-label, randomized (1:2:1)	Fulvestrant + palbociclib + avelumab (F + P + A) (n = 54) vs. fulvestrant + palbociclib (F + P) (n = 111) vs. fulvestrant (F) (n = 55)	mPFS (F + P+A) 8.1 m (HR = 0.75 vs. F, 90% CI 0.50–1.12; *p* = 0.23) mPFS (F + P) 4.6 m (HR = 1.11, 90% CI 0.79–1.55; *p* = 0.62) vs. (F) 4.8 m ORR (F + P+A) 13% (90% CI 5.4–20.5%) vs. (F + P) 9% (90% CI 4.5-13.5%) vs. (F) 7% (90% CI 1.5–13.0)	No new safety signal	NA
NCT03330405/ JAVELIN PARP Medley [14] *Yap TA et al.*	Avelumab	Ib/II	ORR	Single-arm	Talazoparib + avelumab (n = 23)	ORR 34.8% (95% CI 16.4–57.3%) ORR (PD-L1+) 66.7% (95% CI 9.4, 99.2%) ORR (PD-L1-) 25.0% (95% CI 7.3–52.4%)	57%	PD-L1 bDDR tDDR, gDDR TMB bTMB CD8
NCT03608865/ KCSG BR17-04 [15] *Moon YW et al.*	Durvalumab, Tremelimumab	II	ORR, CBR	Single-arm	Durvalumab + tremelimumab (n = 30)	ORR 6.3% CBR (20%)	NA	TMB. TILs PD-L1
*NCT03409198/* *ICON* [16] *Kyte JA et al.*	Nivolumab, Ipilimumab	IIb	Safety, PFS	Open-label, randomized (2:3)	PLD + cyclophosphamide + nivolumab + ipilimumab (n = 49) Vs PLD + cyclophosphamide (n = 33) cross-over to Nivo + Ipi at PD in control arm (n = 16)	mPFS 5.1 m (95% CI 3.4 to 6.5) vs. 3.6 m (95% CI 1.8 to 9.0) HR 0.94 (95% CI 0.59 to 1.51) ORR 55% (E) vs. 48% (C) vs. 19% (cross-over arm)	63% (E) vs. 39% (C) vs. 31% (Ipi + Nivo cross-over)	PD-L1 TMB PBMCs Treg gene expression signature
NCT04061863/ KORNELIA [17] *Kim SH et al.*	Nivolumab	Ib/II	6-month PFS rate	Single-arm	Eribulin + nivolumab (n = 45)	mOS 17.9 m (95% CI: 15.1–NR) mPFS 5.6 m (95% CI, 5.3–7.4) ORR 53.3% (95% CI 37.9–68.3)	65.6%	NA
NCT03430479 [18] *Takada M et al.*	Nivolumab	Ib/II	ORR of unirradiated lesions	Single-arm	RT + nivolumab (n = 28, HR+ = 18)	ORR 11% (90% CI 4–29%) mPFS 4.1 m (95% CI 2.1–6.1)	0%	PD-L1
UMIN000036970/NEWFLAME [19] *Masuda J et al.*	Nivolumab	II	ORR	Single-arm	Abemaciclib + letrozole/fulvestrant + nivolumab (n = 17)	ORR (FUL) 54.5% (95% CI, 28.0–78.7) ORR (LET) 40% (95% CI, 11.7 to 76.9)	100% (LET) 91.6% (FUL)	PD-L1
UMIN000030242/NEWBEAT [20] *Ozaki Y et al.*	Nivolumab	II	ORR	Single-arm	Paclitaxel + bevacizumab + nivolumab (n = 39)	mPFS 16.1 m ORR 74%	53% G3 5% G4.	PD-L1 Serum VEGF-A concentrations
NCT03044730 [21] *Shah AN et al.*	Pembrolizumab	II	PFS	Single-arm, compared with historical control	Capecitabine + pembrolizumab (n = 14 HR+)	mPFS 5.1 m (95% CI, 2.0–11.0) vs. 3 m	NA	PD-L1 TIL score
NCT03222856/KELLY [22] *Pérez-García JM et al.*	Pembrolizumab	II	CBR lasting for ≥24 weeks	Single-arm	Eribulin + pembrolizumab (n = 44)	CBR 56.8 (95% CI, 41.0–71.7) ORR 40.9% (95% CI, 26.3–56.8) mPFS 6 m (95% CI, 3.7–8.4)	56.8% G3 15.9% G4	PD-L1
*NCT03051672* [23] *Barroso-Sousa R et al.*	Pembrolizumab	II	ORR outside the field of radiation	Single-arm	RT + pembrolizumab (n = 8)	ORR: 0%. mOS 2.9 m (95% CI, 0.9–3.6) mPFS 1.4 m (95% CI, 0.4–2.1)	NA	PD-L1 TILs TMB
*NCT02778685* [24] *Yuan Y et al.*	Pembrolizumab	I/II	ORR	Single-arm	Palbociclib + letrozole + pembrolizumab (n = 20)	ORR: 55% (CR 25% PR 30%) mOS 36.9 months (95% CI, 36.9-NR) mPFS 25.2 months (95% CI, 5.3-NR)	87% G3 30% G4	PD-L1 TILs TMB PBMCs
NCT03732391/PEMBRACA * [25] *Cortesi L et al.*	Pembrolizumab	II	ORR	Single-arm	Carboplatin + pembrolizumab (n = 16)	ORR 47%	22.7%	NA
NCT03051659 [7] *Tolaney SM et al.*	Pembrolizumab	II	PFS	Open-label, randomized (1:1)	Eribulin + pembrolizumab (n = 44) vs. eribulin (n = 44)	mPFS 4.1 m (95% CI, 3.5–6.2) vs. 4.2 m (95% CI, 3.7–6.1) mOS 13.4 m (95% CI, 10.4-NA) vs. 12.5 m (95% CI, 8.6-NA) ORR 27% (95% CI, 14.9–42.8%) vs. 34% (95% CI, 20.5–49.9%)	68% (E) vs. 61% (C)	PD-L1 TILs TMB NLR genomic alterations
*NCT02752685* [26] *Novik Y et al.*	Pembrolizumab	II	Best overall response rate	Single-arm	Nab-paclitaxel+ pembrolizumab (n = 20)	ORR: 25% (PR 25%) mOS 15.7 m mPFS 5.6 m	70%	NA
NCT04251169/TATEN Trial (SOLTI1716) [27] *Prat A et al.*	Pembrolizumab	II	ORR	Single-arm	Pembrolizumab + paclitaxel (n = 25)	ORR 53.3% (95% CI 26.6–78.7%) mPFS 7.5 m (95% CI: 5.6–10.2)	53.5%	PD-L1 (IHC) TILs PAM50
NCT02990845 [28] *Chen I-C et al.*	Pembrolizumab	II	8-month PFS rate	Single-arm	Pembrolizumab + GnRH-agonist + exemestane (n = 15)	8 m-PFS rate 64.3% mPFS 10.34 m ORR 35.7% mOS 39.56 m	NA	TILs PD-L1 (IHC) TMB RNAseq IO360 analysis

* enrolled patients with germline BRCA1/2 mutations. TRAEs = treatment-related adverse events, HR+ = hormone-receptor-positive, ORR = overall response rate, mPFS = median progression-free survival, CI = confidence interval, PD-L1 = programmed death-ligand 1, NA = not available, PARP = poly-ADP ribose polymerase, DDR = DNA damage response, bDDR = blood DDR, tDDR = tumor DDR, gDDR = germline DDR, TMB = tumor mutational burden, bTMB = blood TMB, CD = cluster of differentiation, CBR = clinical benefit rate, TILs = tumor-infiltrating lymphocytes, PLD = pegylated liposomal doxorubicin, Nivo = nivolumab, Ipi = ipilimumab, PD = progressive disease, HR = hazard ratio, (E) = experimental, (C) = control, PBMCs = peripheral blood mononuclear cells, Treg = T regulatory, mOS = median overall survival, NR = not reached, RT = radiotherapy, (FUL) = fulvestrant, (LET) = letrozole, VEGF-A = vascular endothelial growth factor-A, NLR = neutrophil-to-lymphocyte ratio, PR = partial response, IHC = immunohistochemistry, GnRH = gonadotropin-releasing hormone.

**Table 2:** Phase II and III clinical trials evaluating ICIs in HR+/HER2− early breast cancer patients.

*Study ID/Name*	Drug	Phase	Primary Endpoint	Study Design	Interventions (No. of pts)	Efficacy Outcomes	TRAEs G ≥ 3	Biomarkers
*NCT03802604/* *SOLTI-1503 PROMETEO* [29] *Pascual T et al.*	Atezolizumab	II	RCB-0/I-rate	Single-arm	T-VEC + atezolizumab (n = 20)	RCB-0/1 rate 25% (95% CI 10.7–44.9%) pCR rate 30%	3.5%	Gene expression TILs PD-L1 TMB
*NCT01042379/* *I-SPY2* [30,31] *Pusztai L et al.* *Nanda R et al.*	Durvalumab	II/III	pCR-rate	Platform trial, non-randomized	Durvalumab + olaparib + paclitaxel → AC (HER2− n = 52) vs. paclitaxel → AC (HER2− n = 157)	pCR 28% (95% CI 18–38%) vs. 14% (95% CI 9–19%)	56% (E) vs. 34% (C)	PD-1 PD-L1 T-cell signature B-cell signature Dendritic-cell signature Mast-cell signature CD68 TIS signature STAT1 signature TAMsurr_TcClassII ratio signature PARPi7 signature Mitotic signatures ESR1_PGR average.
	Pembrolizumab			Open-label, adaptive platform trial	Pembrolizumab paclitaxel → AC (n = 40) vs. paclitaxel → AC (n = 92)	pCR rate 30% (95% CI 17–43%) vs. 13% % (95% CI 7–19%)	42% (E) vs. 18% (C)	NA
*NCT03356860/* *B-IMMUNE* [32] *Devaux A*	Durvalumab	Ib/II	pCR	Single-arm, compared with historical control	Paclitaxel → durvalumab + ddEC (n = 24)	pCR rate: 20%	14.6%	PD-L1 TILs
*NCT04659551/* *GIADA* [33] *Dieci MV*	Nivolumab	II	pCR rate	Single-arm	EC + triptorelin → nivolumab + exemestane (n = 43)	pCR rate: 16.3% (95% CI 7.4–34.9) RCB 0-I rate: 25.6% (95% CI, 14.0–41.8)	CT phase: 23.4% Nivolumab phase: 52.9%	TILs Immune-related gene expression signatures Specific immune cell subpopulations PAM50
*NCT04075604/* *CheckMate7A8* [34] *Jerusalem G et al.*	Nivolumab	Ib/II	pCR rate	Single-arm	Nivolumab + palbociclib + anastrozole (n = 21)	pCR rate 9.5% ORR 71.4%	81%	NA
*NCT04109066/* *Checkmate 7FL* [35] *Loi S et al.*	Nivolumab	III	pCR rate	Randomized (1:1), placebo-controlled	Nivolumab + paclitaxel → A/EC or ddA/EC → adjuvant nivolumab plus endocrine therapy (n = 263) vs. placebo + paclitaxel → A/EC or ddA/EC (n = 258)	pCR rate: 24.5% (95% CI 19.4–30.2) vs. 13.8% (95% CI 9.8–18.7) AD: 10.5% (OR 2.05; 95% CI 1.29–3.27; *p* = 0.0021) RCB 0-I rate: 30.7% (95% CI 25.2–36.8) vs. 21.4% (95% CI 16.5–26.9)	35% (E) vs. 32% (C)	PD-L1
*NCT03725059/* *KEYNOTE-756* [36] *Cardoso F et al.*	Pembrolizumab	III	pCR, EFS	Randomized (1:1), placebo-controlled	Pembrolizumab + paclitaxel → A/EC or ddA/EC → pembrolizumab post-surgery + endocrine therapy (n = 635) vs. placebo + paclitaxel → A/EC or ddA/EC (n = 643)	pCR rate: 24.3% vs. 15.6%	52.5% (E) vs. 46.4% (C)	PD-L1 TILs

TRAEs = treatment-related adverse events, RCB = residual cancer burden, T-VEC = talimogene laherparepvec, CI = confidence interval, pCR = pathologic complete response, TILs = tumor-infiltrating lymphocytes, PD-L1 = programmed death-ligand 1, TMB = tumor mutational burden, AC = adriamycin + cyclophosphamide, (E) = experimental, (C) = control, PD-1 = programmed death-1, CD = cluster of differentiation, STAT1 = signal transducer and activator of transcription 1, PARPi = poly-ADP ribose polymerase inhibitor, ESR1 = estrogen receptor 1, PGR = progesterone receptor, NA = not available, RT = radiotherapy, T = tumor, dd = dose dense, MHC-I = major histocompatibility complex-1, EC = epirubicin + cyclophosphamide; SBRT = stereotactic beam radiation therapy; CI = confidence interval, CT = chemotherapy, AD = absolute difference.

**Table 3:** Ongoing phase II and III clinical trials evaluating checkpoint inhibitors and their combination therapies in HR+ breast cancer.

Trial ID/Name	Phase	Estimated Enrollment	Estimated End Date	Study Design	Population	ICI	Combination Agent(s)	Primary Endpoint(s)
***Advanced setting***								
NCT05187338	I/II	100	October 2024	Single group assignment, open-label	Advanced solid tumors	Ipilimumab	Pembrolizumab + durvalumab	Safety PFS DCR DOR
NCT03650894	II	138	April 2026	Single group assignment, open-label	HER2− LA unresectable mBC (for TNBC, AR+ was required)	Ipilimumab	Nivolumab, bicalutamide	Best ORR PFS 2y-OS rate
NCT05620134	I/II	149	February 2026	Non-randomized open-label	Advanced solid tumors	JK08 **	NA	DLT Dose finding Safety and tolerability
NCT04683679	II	56	January 2025	Non-randomized, open-label	Advanced HER2− BC	Pembrolizumab	Olaparib + radiation	ORR
NCT06312176	III	1200	April 2031	Randomized, open-label	Unresectable LA or HR+/HER2− mBC	Pembrolizumab	Sacituzumab–tirumotecan	PFS
NCT04448886	II	110	June 2027	Randomized, open-label	Unresectable LA or HR+/HER2− mBC	Pembrolizumab	Sacituzumab–govitecan	PFS
***Early setting***								
NCT03573648/ImmunoADAPT	II	33	December 2025	Randomized, open-label	Stage II-III HR+/HER2−	Avelumab	Palbociclib Endocrine therapy	CCR
NCT04243616	II	36	January 2025	Single group assignment, open-label	Stage II-III HER2− BC with positive PD-L1 and/or PD-L1 protein expression *	Cemiplimab	Paclitaxel, carboplatin, doxorubicin, cyclophosphamide	pCR
NCT01042379/I-SPY	II	5000	December 2031	Randomized, open-label	Stage II or III, or T4 M0, inflammatory cancer or regional stage IV ^§^ BC of any subtype	Cemiplimab	Chemotherapy	pCR for each biomarker signature established at trial entry
NCT06058377	III	3680	May 2026	Randomized, open-label	Stage II-III HR+/HER2− BC MP2	Durvalumab	Paclitaxel Doxorubicin Cyclophosphamide	BC-EFS
NCT01042379/I-SPY	II	5000	December 2031	Randomized, open-label	Stage II or III, or T4, M0, inflammatory cancer or regional stage IV ^§^, gBRCA1/2 mut, HER2− BC	Durvalumab	Olaparib	pCR for each biomarker signature established at trial entry
NCT06112379/TROPION-Breast04	III	1728	August 2030	Randomized open-label	Stage II or III TNBC or HR-low/HER2− BC	Durvalumab	Dato-DXd, adjuvant chemotherapy (if residual disease)	EFS
NCT03815890/BELLINI	II	80	January 2033	Non-randomized platform study	Resectable stage I-III BC (TN or luminal B)	Nivolumab	Ipilimumab	pCR rate per cohort
NCT05203445	II	23	January 2026	Single group assignment, open-label, non-randomized	Stage I-III, HER2− BC with gBRCA1, gBRCA2, gPALB2, gRAD51C, or gRAD51D mut	Pembrolizumab	Olaparib	Pathologically negative MRI-guided biopsy
NCT02971748	II	37	December 2024	Single group assignment, open-label	HR+ localized inflammatory BC who did not achieve a pCR	Pembrolizumab	Endocrine therapy, radiation	DFS
NCT02957968	II	47	February 2025	Non-randomized, open-label	Stage II-III HER2− BC	Pembrolizumab	Decitabine, followed by cyclophosphamide, paclitaxel, carboplatin	Percentage of increase in tumor and stroma with TILs from baseline pretreatment biopsy to post-immunotherapy biopsy following administration of decitabine followed by pembrolizumab
NCT04443348	II	120	December 2024	Randomized, open-label	Stage II-III HER2− BC, for HR+: histologic G2-3 or a high-risk genomic assay score	Pembrolizumab	Radiation therapy boost, paclitaxel, carboplatin, cyclophosphamide, doxorubicin, capecitabine	TILs, CD3+/CD8+ T-cell Breast Immunoscore Rate of pathologic response in the lymph node

* Positivity was defined as PD-L1 and/or PD-L2 expression of ≥1% of immune cells within the stroma or in cancer cells. ** CTLA-4 targeting IL-15 antibody fusion protein. ^§^ Where supraclavicular lymph nodes are the only site of metastasis. ICI: immune checkpoint inhibitor, PFS: progression-free survival, DCR: disease control rate, DOR: duration of response, HER2− = HER2-negative, LA = locally advanced, mBC = metastatic breast cancer, TNBC = triple-negative breast cancer, AR+ = androgen-receptor-positive, ORR: overall response rate, DLT: dose-limiting toxicity, 2y: 2 years, BC = breast cancer, HR = hormone receptor, HR+ = hormone-receptor-positive, CCR: clinical response rate, PD-L1 = programmed death-ligand 1, pCR = pathologic complete response, MP2 = Mammaprint ultra-high, EFS: event-free survival, Dato-DxD = datopotamab–deruxtecan, gBRCA1/2 mut = germinal BRCA1/2 mutated, MRI: magnetic resonance imaging, DFS: disease-free survival, TILs: tumor-infiltrating lymphocytes, CD: cluster of differentiation, CTLA-4 = cytotoxic T-lymphocyte antigen-4.
